# The Early Activation of CD8^+^ T Cells Is Dependent on Type I IFN Signaling following Intramuscular Vaccination of Adenovirus Vector

**DOI:** 10.1155/2014/158128

**Published:** 2014-05-27

**Authors:** Masahisa Hemmi, Masashi Tachibana, Sayaka Tsuzuki, Masaki Shoji, Fuminori Sakurai, Kenji Kawabata, Kouji Kobiyama, Ken J. Ishii, Shizuo Akira, Hiroyuki Mizuguchi

**Affiliations:** ^1^Laboratory of Biochemistry and Molecular Biology, Graduate School of Pharmaceutical Sciences, Osaka University, 1-6 Yamadaoka, Suita, Osaka 565-0871, Japan; ^2^Laboratory of Regulatory Sciences for Oligonucleotide Therapeutics, Clinical Drug Development Unit, Graduate School of Pharmaceutical Sciences, Osaka University, 1-6 Yamadaoka, Suita, Osaka 565-0871, Japan; ^3^Laboratory of Stem Cell Regulation, National Institute of Biomedical Innovation, 7-6-8 Asagi, Saito, Ibaraki, Osaka 567-0085, Japan; ^4^Laboratory of Adjuvant Innovation, National Institute of Biomedical Innovation, 7-6-8 Asagi, Saito, Ibaraki, Osaka 567-0085, Japan; ^5^Laboratory of Vaccine Science, World Premier International Research Center Immunology Frontier Research Center, Osaka University, 3-1 Yamadaoka, Suita, Osaka 565-0871, Japan; ^6^Laboratory of Host Defense, World Premier International Research Center Immunology Frontier Research Center, Osaka University, 3-1 Yamadaoka, Suita, Osaka 565-0871, Japan; ^7^Department of Host Defense, The Research Institute for Microbial Diseases, Osaka University, 3-1 Yamadaoka, Suita, Osaka 565-0871, Japan; ^8^iPS Cell-Based Research Project on Hepatic Toxicity and Metabolism, Graduate School of Pharmaceutical Sciences, Osaka University, 1-6 Yamadaoka, Suita, Osaka 565-0871, Japan; ^9^Laboratory of Hepatocyte Differentiation, National Institute of Biomedical Innovation, 7-6-8 Asagi, Saito, Ibaraki, Osaka 567-0085, Japan; ^10^The Center for Advanced Medical Engineering and Informatics, Osaka University, 2-2 Yamadaoka, Suita, Osaka 565-0871, Japan

## Abstract

Few of the vaccines in current use can induce antigen- (Ag-) specific immunity in both mucosal and systemic compartments. Hence, the development of vaccines that realize both mucosal and systemic protection against various pathogens is a high priority in global health. Recently, it has been reported that intramuscular (i.m.) vaccination of an adenovirus vector (Adv) can induce Ag-specific cytotoxic T lymphocytes (CTLs) in both systemic and gut mucosal compartments. We previously revealed that type I IFN signaling is required for the induction of gut mucosal CTLs, not systemic CTLs. However, the molecular mechanism via type I IFN signaling is largely unknown. Here, we report that type I IFN signaling following i.m. Adv vaccination is required for the expression of type I IFN in the inguinal lymph nodes (iLNs), which are the draining lymph nodes of the administration site. We also showed that the type I IFN signaling is indispensable for the early activation of CTLs in iLNs. These data suggested that type I IFN signaling has an important role in the translation of systemic innate immune response into mucosal adaptive immunity by amplifying the innate immune signaling and activating CTLs in the iLN.

## 1. Introduction

Mucosal membranes have enormous surface areas, through which most pathogens access the body, and therefore, they are important in vaccine development to establish protective immune responses at mucosal sites as well as systemic sites [1, 2]. Hence, the development of vaccines that realize both mucosal and systemic protection against various pathogens is a high priority in global health. However, few of the vaccines in current use can induce antigen- (Ag-) specific immunity in both mucosal and systemic compartments [3]. In general, the induction of mucosal immunity by systemic administration of vaccine has proven to be difficult due to the unique immunological features of the mucosal immune system [3].

The replication incompetent recombinant adenovirus vector (Adv) has several advantages as a gene therapy vector: it provides the highest gene transduction efficiency among the currently available vectors, it has low genotoxicity because it is not integrated into the chromosomal DNA, and it can be easily prepared in high titers. Moreover, it has been revealed that Adv can be applied to gene therapy-based vaccines, and several Adv and vaccine protocols have been used in preclinical studies [4]. Recently, it has been reported that intramuscular (i.m.) immunization with an Adv vaccine-expressing simian immunodeficiency virus (SIV) gag can induce functional and sustainable SIV gag-specific cytotoxic T lymphocytes (CTLs) in the gut mucosal compartments as well as the systemic compartments in mice and rhesus macaques [5–7]. Adv is expected to become a next generation mucosal vaccine that combats severe intracellular pathogens [8].

Innate immune responses have been clearly shown to be critical for the optimal induction of adaptive immune responses [9–11]. Moreover, there is accumulating evidence that the adjuvants which activate innate immunity are effective for the induction of vaccine effects [12]. Several studies have revealed that Adv-derived nucleic acids, adenoviral genomic DNA, and adenoviral noncoding RNA (virus-associated RNA (VA-RNA)) activate innate immunity and produce innate immune cytokines. The adenoviral genomic DNA triggers innate immune responses through several pattern recognition receptors and adaptor molecules, such as Toll-like receptor 9 (TLR9)/myeloid differentiation primary-response protein 88 (MyD88) [13–15], and cGAMP synthase (cGAS)/stimulator of interferon genes (STING) [16], and induces the production of type I IFNs and proinflammatory cytokines. VA-RNA also induces the production of type I IFN through IFN-*β* promoter stimulator-1 (IPS-1) [17]. Type I IFN induced by Adv immunization has been shown to have an important role in the subsequent systemic adaptive immunity. It is indicated that not only dendritic cells (DCs), but also other types of cells, such as stromal cells, produce IFN-*β in vivo* and are involved in the induction of adaptive immunity [17–19]. Thus, determining the role of IFN-*β in vivo *is important for vaccine development. Moreover, the magnitudes of type I IFN correlate with the titers of Ad-specific neutralizing antibodies, suggesting that type I IFN signaling controls the efficacy of Adv vaccine [20]. We previously reported that type I IFN signaling following i.m. Adv vaccination is required for the induction of Ag-specific CTLs not in the systemic compartment but in the gut mucosal compartment [8]. Thus, type I IFN is important for the positive regulation of the Ag-specific gut mucosal cellular immune response. However, it is unclear how the Adv-induced type I IFN signaling translates innate immune response into gut mucosal adaptive immunity.

In this study, we report that type I IFN signaling is indispensable for the expression of IFN-*α*, IFN-*β*, cGAS, and TLR9in the draining lymph nodes (DLNs) in the early stage following i.m. Adv vaccination. Moreover, we found that type I IFN signaling is essential for the early activation of CD8^+^ T cells in the DLNs. These data suggested that type I IFN signaling has an important role in the translation of systemic innate immunity into mucosal adaptive immunity. Our findings should lead to the development of safer and more efficient mucosal Adv vaccines.

## 2. Materials and Methods

### 2.1. Mice

C57BL/6J (wild-type, WT) mice were purchased from Japan SLC (Hamamatsu, Japan) and IFNAR2^−/−^mice (C57BL/6J background) were established as described previously [21]. All mice were housed in an animal facility under specific pathogen-free conditions and used at 7-8 weeks of age. All animal experimental procedures used in this study were performed in accordance with the institutional guidelines for animal experiments at Osaka University and the National Institute of Biomedical Innovation.

### 2.2. Adv Production and Immunization

The adenovirus type 5 vector-expressing LacZ (Ad-LacZ) was constructed as described previously [22]. Briefly, the expression cassette containing the chicken *β*-actin promoter with the cytomegalovirus enhancer (CA) driven [23] LacZ gene was inserted into the E1/E3-deleted adenovirus type 5 genome. This virus was grown in 293 cells using standard techniques. Ad-LacZ was purified with CsCl_2_ step gradient ultracentrifugation, dialyzed with a solution containing 10 mM Tris (pH 7.5), 1 mM MgCl_2_, and 10% glycerol, and stored in aliquots at −80°C. Determination of the virus particle (vp) titers was accomplished spectrophotometrically according to the methods of Maizel et al. [24]. All mice were injected under anesthesia in the right and left quadriceps muscles with Ad-LacZ (5 × 10^9^ vp per muscle; total 10^10^ vp per mouse).

### 2.3. Isolation of Mononuclear Cells

The inguinal lymph nodes and mesenteric lymph nodes were dissected and pressed through a 70 *μ*m cell strainer. The cells were washed with FACS buffer (2% FCS, 0.02% sodium azide in PBS).

### 2.4. DNA Isolation and qPCR

Total DNA was isolated from whole tissues using a DNeasy Blood & Tissue Kit (QIAGEN). Quantitative PCR was performed with Taqman Fast Universal PCR Master Mix (Applied Biosystems) using an Applied Biosystem StepOnePlus Real-Time PCR System. Absolute quantities were calculated using standard curves. The copy numbers of each gene were normalized with those of GAPDH. The primer sequences in this study are Gapdh forward, 5′-CAATGTGTCCGTCGTGGATCT-3′; Gapdh reverse, 5′-GTCCTCAGTGTAGCCCAAGATG-3′; Ad E4 forward, 5′-GGGATCGTCTACCTCCTTTTGA-3′; Ad E4 reverse, 5′-GGGCAGCAGCGGATGAT-3′.

### 2.5. RNA Isolation and RT-PCR

Total RNA was isolated from mononuclear cells using ISOGEN (Nippon Gene). cDNA was synthesized using 400 ng of total RNA with a Superscript VILO cDNA Synthesis Kit (Invitrogen) according to the manufacturer's instructions. Quantitative RT-PCR was performed with THUNDERBIRD qPCR Mix (TOYOBO) using an Applied Biosystem StepOnePlus Real-Time PCR System. Relative expression was calculated using the  ΔΔC_T_  method. The mRNA level of each gene was normalized with that of *β*-actin. The primer sequences used in this study are* Actb* forward, 5′-GGCTGTATTCCCCTCCATCG-3′;* Actb* reverse, 5′-CCAGTTGGTAACAATGCCATGT-3′;* Ifna* forward, 5′-CTTCCACAGGATCACTGTGTACCT-3′;* Ifna* reverse, 5′-TTCTGCTCTGACCACCTCCC-3′;* Ifnb* forward, 5′-CTGGAGCAGCTGAATGGAAAG-3′;* Ifnb* reverse, 5′-CTTCTCCGTCATCTCCATAGGG-3′;* Mb21d1* forward, 5′-AGGAAGCCCTGCTGTAACACTTCT-3′;* Mb21d1* reverse, 5′-AGCCAGCCTTGAATAGGTAGTCCT-3′;* Tlr9* forward, 5′-ATGGTTCTCCGTCGAAGGACT-3′;* Tlr9* reverse, 5′-GAGGCTTCAGCTCACAGGG-3′;* Ddx41* forward, 5′-AGTCCGCCAAGGAAAAGCAA-3′;* Ddx41* reverse, 5′-CTCAGACATGCTCAGGACATAAC-3′;* Il1b* forward, 5′-GCAGCAGCACATCAACAAG-3′;* Il1b* reverse, 5′-CGGGAAAGACACAGGTAGC-3′;* Tnfa* forward, 5′-CCCTCACACTCAGATCATCTTCT-3′;* Tnfa* reverse, 5′-GCTACGACGTGGGCTACAG-3′.

### 2.6. Flow Cytometry

The antimouse antibodies used in this study were purchased from eBioscience (PE-Cy7-CD3*ε* (145-2C11)) and BioLegend (FITC-CD4 (GK1.5), APC-Cy7-CD8*α* (53-6.7), Pacific Blue-CD69 (H1.2F3)). Flow cytometry analysis was performed using a fluorescence-activated cell sorting (FACS) LSR Fortessa flow cytometer and BD FACSDiva software (BD Bioscience). Dead cells were excluded by 7-amino-actinomycin D staining (eBioscience).

### 2.7. Statistics

All results are shown as the mean ± standard error of the mean. Statistical significance was analyzed by the One-way ANOVA among groups.

## 3. Results

### 3.1. Adv Was Mainly Distributed to the Inguinal Lymph Nodes following i.m. Adv Vaccination

**Figure 1:**
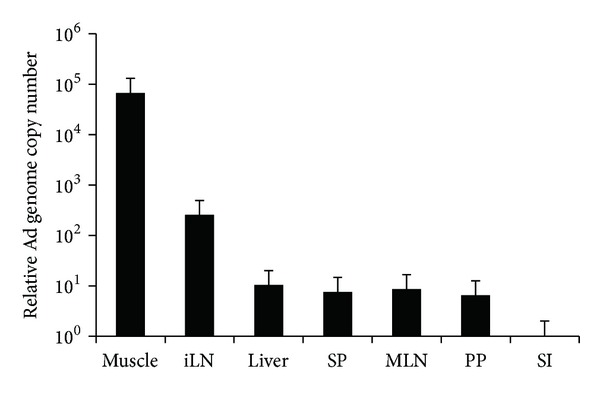
The tissue distribution of Adv following i.m. Adv vaccination. At 8 hours after the i.m. vaccination of 10^10^ vp of Ad-LacZ, the tissue distribution of Adv was determined by the absolute quantity of Ad E4 gene in each tissue, normalized by the copy number of GAPDH. The graphs represent the relative Ad genome copy number in each tissue normalized by that of the small intestine. Data are shown as the means ± S.E.M. (*n* = 3). iLN, inguinal lymph node; SP, spleen; MLN, mesenteric lymph node; PP, Peyer's patch; SI, small intestine.

Innate immune responses are elicited within several hours after i.m. Adv vaccination. To reveal the sites where Adv induces the responses, we first examined Ad genome copy numbers in each tissue at 8 hours after i.m. Adv vaccination. Adv was mainly distributed to the muscles and inguinal lymph nodes (iLNs), the DLNs of the vaccination site (Figure 1). On the other hand, Adv was barely distributed to mesenteric lymph node (MLN), which is important for gut mucosal immunity. The distributions of Adv in the liver, spleen (SP), Peyer's patches (PP), and small intestine (SI) were similar to those in the MLN. These results suggested that Adv should induce innate immune responses in the iLNs.

### 3.2. Type I IFN Signaling Enhances the Expression of Innate Immune Cytokines and DNA Sensors in the Draining Lymph Nodes

**Figure 2:**
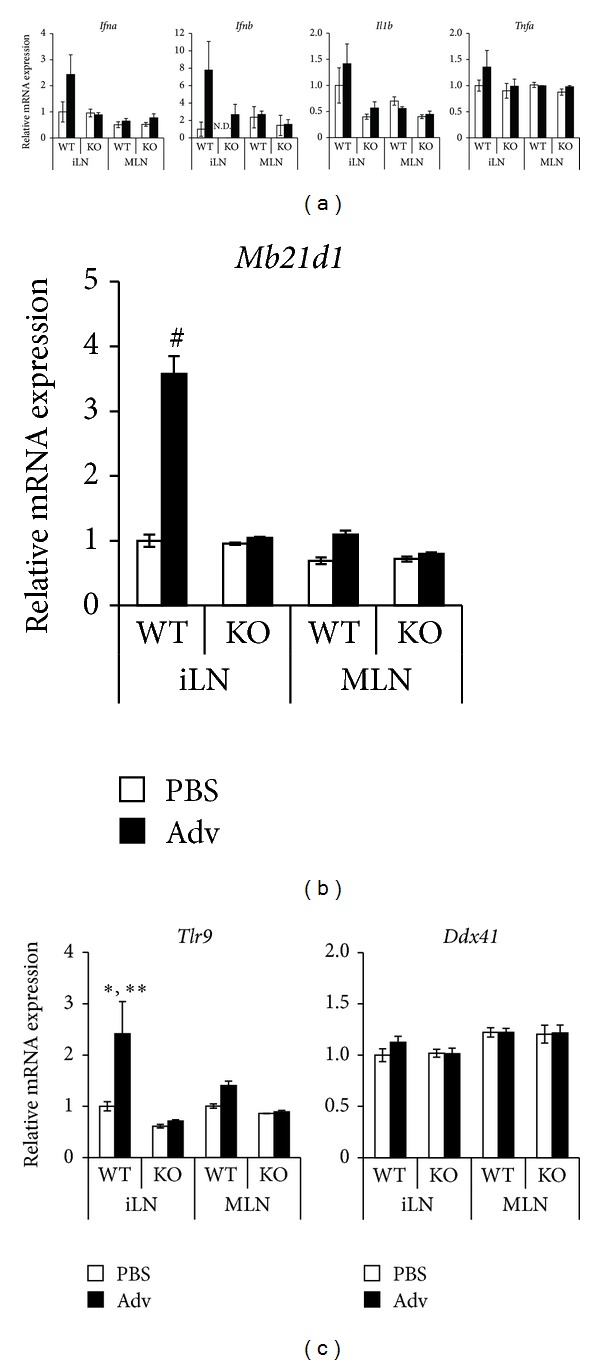
Relative mRNA expressions in the iLNs and MLN of WT and IFNAR2 KO mice following i.m. Adv vaccination. At 8 hours after the i.m. vaccination of 10^10^ vp of Ad-LacZ, total RNA was extracted from mononuclear cells in the LNs of each mouse. The mRNA expressions of* Ifna*,* Ifnb, Il1b, Tnfa *(a),* Mb21d1 *(b),* Tlr9*, and* Ddx41 *(c) in the LNs were measured by qRT-PCR, normalized by* Actb*. The graphs represent the relative mRNA expression of each gene normalized by that of PBS-administrated WT mice. Data are shown as the means ± S.E.M. (*n* = 3) and are representative of two independent experiments.  *P < 0.05 compared with other groups except for the MLN of Adv-administrated WT mice.  **P < 0.01 compared with the iLNs of IFNAR2 KO mice. ^#^ *P* < 0.0001 compared with other groups.

Next, to reveal the roles of type I IFN signaling in the induction of gut mucosal CTLs, we examined the expression of innate immune cytokines and interferon-stimulated genes (ISGs) in iLNs and MLN by using type I IFN receptor knockout (IFNAR2 KO) mice, which have defects in immune responses to viruses and double-stranded DNA. In Adv-administrated WT mice, the expression of IFN-*α* and IFN-*β* was upregulated in the iLNs, where Adv was mainly distributed at this time point (Figure 2(a)). Moreover, in Adv-administrated WT mice, the expression of IL-1*β* and TNF-*α* tended to be upregulated in the iLNs (Figure 2(a)). On the other hand, the expression of these cytokines was not upregulated in the MLN, where Adv was barely distributed. Similarly, the expression of cGAS, a representative ISG, that is, encoded by* Mb21d1* [25], was upregulated in the iLNs (Figure 2(b)). However, in Adv-administrated IFNAR2 KO mice, the expression of IFN-*α*, IFN-*β*, and cGAS in the iLNs was not upregulated following i.m. Adv vaccination. In addition, in Adv-administrated IFNAR2 KO mice, the expression of IL-1*β* and TNF-*α* in the iLNs was lower than that in the iLNs of Adv-administrated WT mice.

Since cGAS is one of the DNA sensors detecting Ad genome [16], we next examined the expression of other DNA sensors, TLR9 and DDX41, which are widely known for sensing Ad genome [15, 26]. In Adv-administrated WT mice, only the expression of TLR9 was upregulated in the iLNs (Figure 2(c)). However, it was not upregulated in the iLNs of Adv-administrated IFNAR2 KO mice. These data suggested that type I IFN signaling enhances the expression of IFN-*α*, IFN-*β*, cGAS, and TLR9 in the iLNs, where much Adv is distributed from the muscles. It is speculated that the lack of type I IFN signaling in the innate immune responses at the iLNs leads to the significant reduction of antigen-specific CTLs in the gut mucosal compartment.

### 3.3. Type I IFN Signaling Is Required for the Early Activation of CTLs in iLNs

**Figure 3:**
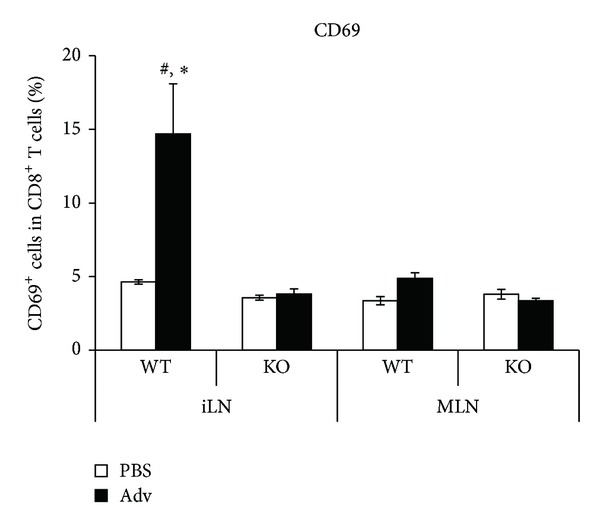
The frequencies of early activated CD8^+^ T cells in the iLNs and MLN of WT and IFNAR2 KO mice following i.m. Adv vaccination. At 24 hours after the i.m. vaccination of 10^10^ vp of Ad-LacZ, the frequencies of CD69^+^ T cells in CD8^+^ T cells in the LNs of each mouse were measured by flow cytometry. Data are the pools of three independent experiments and are shown as the means ± S.E.M. (*n* = 6).  *P < 0.001  compared with the iLNs of PBS-administrated WT mice and MLN of Adv-administrated WT mice. ^#^ *P* < 0.0001 compared with other groups.

Since type I IFN signaling was not induced in IFNAR2 KO mice following i.m. Adv vaccination, we hypothesized that the early activation of T cells is not elicited sufficiently in IFNAR2 KO mice. After antigenic stimulation, T cells express a series of several activation markers, including CD69, CD44, and CD25, dependent on the developmental stages from naïve to effector. To examine whether the type I IFN signaling following i.m. Adv vaccination has an effect on an early activation marker, CD69 [27, 28], on CD8^+^ T cells, we estimated the frequencies of CD69^+^ cells in CD8^+^ T cells residing in the DLNs. In Adv-administrated WT mice, the frequencies of CD69^+^ cells in CD8^+^ T cells in the iLNs were increased, while those in the MLN were not. However, in the case of Adv-administrated IFNAR2 KO mice, the frequencies of CD69^+^ cells in CD8^+^ T cells in the iLNs were significantly reduced compared with those of Adv-administrated WT mice (Figure 3). Thus, these data correlate with the results shown in Figure 2, in which the type I IFN response was elicited at the iLNs and diminished in IFNAR2 KO mice. These results indicated that type I IFN signaling induces the expression of CD69 on CD8^+^ T cells in Adv-administrated mice. Collectively, these data suggest that the early activation of CD8^+^ T cells via type I IFN signaling promotes the induction and/or migration of gut mucosal Ag-specific CTLs.

## 4. Discussion

In this study, we demonstrated that type I IFN signaling following i.m. Adv vaccination promotes the expression of IFN-*α*, IFN-*β*, and cGAS in the iLNs where Adv is mainly distributed at this time point and strongly induces the expression of CD69 on CD8^+^ T cells in the iLNs. The expression of type I IFN is amplified through IFNAR [29, 30]. Therefore, it is reasonable that the expression of type I IFN is decreased in IFNAR2 KO mice. We observed that IFN-*β* expression was more strongly induced by type I IFN signaling than IFN-*α* expression. It has been reported that IFN-*α* is mainly produced by plasmacytoid dendritic cells (pDCs), while IFN-*β* is mainly produced by myeloid DCs (mDCs) and mouse embryonic fibroblasts (MEFs) [17, 31, 32]. Hence, it is speculated that type I IFN signaling contributes to IFN-*β* production from DCs and fibroblasts in the iLNs, such as stromal cells, following i.m. Adv vaccination. In addition, we observed a significant reduction in TLR9 expression as well as cGAS expression in IFNAR2 KO mice. cGAS and TLR9 have been reported to be the DNA sensors responsible for the recognition of adenoviral DNA leading to the induction of type I IFN production [15, 16]. It is speculated that type I IFN signaling promotes the detection of Adv by cGAS and TLR9 and amplifies their signaling* in vivo*.

Recently, Weerd et al. revealed that IFN-*β* binds to the low-affinity component of IFNAR, IFNAR1, in the absence of IFNAR2 [33]. Moreover, IFNAR1-IFN-*β* complex activates unique intracellular signaling. However, in our study, we did not observe such phenomenon in IFNAR2 KO mice. For example, Weerd et al. showed that in IFNAR2 KO peritoneal exudate cells (PECs), IL-1*β* expression was upregulated 13.5-fold by IFN-*β* compared to nontreated IFNAR2 KO PECs. On the other hand, in our study, IL-1*β* expression in the iLNs of Adv-administrated IFNAR2 KO mice was upregulated just only 1.42-fold as much as that of PBS-administrated IFNAR2 KO mice (Figure 2(a)). Thus, it is speculated that the level of IFN-*β* in our study would be much lower than that in their study and the IFN-*β* signaling in our study would be transmitted via IFNAR2, which is the high-affinity component of IFNAR.

We observed a significant reduction of CD69 expression on CD8^+^ T cells in the iLNs of IFNAR2 KO mice following i.m. Adv vaccination. Our results are consistent with a previous report that the expression of CD69 is strongly induced by type I IFN [34, 35]. CD69 inhibits egress of T cells from the spleen and secondary lymphoid tissues during T cell maturation [35]. It is speculated that the reduction of CD69 expression in IFNAR2 KO mice induces egress of CD8^+^ T cells from the iLNs in the early stage of T cell maturation. In consequence, activated CD8^+^ T cells in the iLNs fail to mature sufficiently, and thus, these cells do not acquire a gut-homing capacity. Moreover, Alari-Pahissa et al. recently reported that CD69 does not affect CD8^+^ T cell priming following Ag-expressing vaccinia virus vector immunization [36]. Considering our previous finding that systemic Ag-specific CTLs are induced in IFNAR2 KO mice following i.m. Adv vaccination as similar as WT mice [8], it is likely that the reduction of CD69 expression on CD8^+^ T cells does not alter CD8^+^ T cell priming. For these reasons, it is suggested that type I IFN signaling-induced CD69 expression on CD8^+^ T cells might regulate Ag-specific CTLs in the gut mucosal compartment.

In summary, we have shown the molecular mechanism of the induction of gut mucosal CTLs following i.m. Adv vaccination. We found that type I IFN signaling is required for the production of large amounts of type I IFN and the upregulation of CD69 on CD8^+^ T cells in the iLNs. Our findings should contribute to the development of more efficient and safer mucosal vaccines and adjuvants.
